# Interesting Case of Repair of Tissue after Injury

**Published:** 1884-01

**Authors:** W. T. Becker

**Affiliations:** First Assistant Physician, Milwaukee Insane Asylum, Milwaukee, Wis.


### ARTICLE V.

INTERESTING CASE OF REPAIR OF TISSUE AFTER INJURY. By W. T. Becker, M.D., First Assistant Physician, Milwaukee Insane Asylum, Milwaukee, Wis.

The following case of repair of tissue after an injury I thought of sufficient interest to make known to your readers: On the 16th of June last, Miss. T., æt. 23, while ironing linen at a mangle, caught her right hand between the rollers of the machine, and in rapidly withdrawing it the soft tissue of her middle finger was severed at the second phalangeal joint. The severed portion of finger was lost in the machine, while the denuded bone was still intact. Immediately after the accident I saw the patient. The hæmorrhage was slight, because of the lacerated nature of the injury, the bone was entirely bare down to the head of the first phalanx, the periosteum appeared destroyed at some parts. Later, the severed finger was found; it resembled in shape the finger of a glove, only somewhat thicker, being hollow in the interior, having the nail upon it, and containing part of the tendon of the deep flexor muscle. This glove-like appearance suggested the grafting of it around the bone, which would have been done had its discovery been more timely. Sloughing was anticipated, and the question of amputation presented itself; it was decided to wait to see how much reparation would ensue. Accordingly, the parts were washed in dilute carbolic acid solution, and dressed in vaseline ointment, merely as a protecting dressing.

Reparation began quickly, granulations springing up about the soft end of the remaining digit, and spreading until they reached the top of the bone, which they eventually covered. In the meantime, the pellicle formed and spread, and the epidermis was reproduced, until the finger assumed its original proportions. During the healing process compound tinct. of benzoin and Peruvian balsam (sometimes both, and in varying quantity,) were mixed with the ointment, when the granulation tissue required stimulation.

At first, a dense white tissue occupied the site of the nail, which it was supposed would permanently assume its function. Subsequently, however, a real nail was reproduced. The use of the hands, which in the absence of this result, would have been seriously interfered with (being the right one, and the lady being a pianist,) has suffered little permanent improvement.

This case presents an interest because the nature of the repair assumes more nearly the character of a reproduction. It cannot, however, be considered as such in the comprehensive sense in which that term is applied in surgery, but merely as reparation, not remarkable so much for its extent as its form. Complete reproduction is only found to prevail in the lower organisms, where members of complex structures (even such as the heads of Hydræ) when severed, will be reproduced. The degree of reproduction diminishes as we ascend the scale of organized beings. In the higher animals it becomes only partial, and in the human species its manifestations are at a minimum, and confined only to that group of tissues which develop from the external of the embryonic layers.

---

